# Prevalence of Intestinal Parasitic Infection among Food Handlers in Northwest Iran

**DOI:** 10.1155/2016/8461965

**Published:** 2016-04-03

**Authors:** Davoud Balarak, Mohammad Jafari Modrek, Edris Bazrafshan, Hossein Ansari, Ferdos Kord Mostafapour

**Affiliations:** ^1^Department of Environmental Health, Health Promotion Research Center, Zahedan University of Medical Sciences, Zahedan, Iran; ^2^Department of Parasitology, Infectious Diseases and Tropical Medicine Research Center, Zahedan University of Medical Sciences, Zahedan, Iran; ^3^Department of Epidemiology and Biostatistics, Health Promotion Research Center, Zahedan University of Medical Sciences, Zahedan, Iran

## Abstract

Parasitic diseases are among the most important infectious diseases and pose health problems in many countries, most especially in developing countries. Workers at food centers could transmit parasitic infections in the absence of sanitation. This is a descriptive study conducted to determine the prevalence of intestinal parasitic infections in food clerks in the city of Tabriz in 2014. Data was recorded in the offices of the health center for all food handlers who were referred to the laboratory for demographic and stool tests to receive the health card. Parasitic infection was observed in 172 cases (3.73%) of 4612 samples. A total of 156 positive samples (90.69%) were related to protozoa and 16 (9.3%) were related to helminthes. Most of the parasitic infections were related to* Giardia* and* Entamoeba coli* and the lowest infection was related to* H. nana*. Also, there was a significant relationship between level of education and parasitic infection rate (*P* = 0.0044). But there was no significant difference between the type of infection and amount of intestinal parasites. The results show that the prevalence of intestinal parasites, especially pathogenic protozoa, is common in some food handlers. Therefore, more sanitary controls are required and increasing of education will play a crucial role in improving the health of these people.

## 1. Introduction

The parasitic infections are considered as one of the major health problems in the world and especially in developing countries [1, 2]. According to the World Health Organization (WHO), nearly two-thirds of the world is infected with one kind of intestinal parasite and* Ascaris and Giardia* infections have the highest rate among all kinds [3]. Due to geographical location, climate, extent of the area, and cultural and biological characteristics, there is a suitable environment for the activity of various parasites in Iran [4, 5]. The reasons for high incidence of parasites in some parts of the country are a result of specific climate of the regions, local customs, and the use of human and animal fertilizers in agriculture and olericulture [6]. Lack of clean and safe water, high population density, lack of proper disposal of waste, noncompliance with health standards (social and individual), lack of adequate washing of vegetables, and lack of well cooked meat lead to high prevalence of intestinal parasites [7, 8]. Studies in different parts of Iran revealed that there is infection of intestinal parasites between studied groups. For this reason, several studies have been conducted on the prevalence of parasitic infections in different parts of the country that show the prevalence of parasitic infections between 2 and 61% in studied populations [9–11]. According to pervious researches, the prevalence level of parasitic diseases was as follows: Kermanshah (59.13%) [3], Mazandaran (21%) [12], Kashan (46.9%) [13], and Ardabil (27.7%) [14], while it was 13.7% and 8.4% for Semnan [15] and Ghaemshahr [16] that indicates the great prevalence of these infections from the statistical viewpoint. As a result of the high incidence of parasitic infection in the country, the identification of infection, knowing the methods of transmission, and preventing their transmission are of paramount importance and are considered as health priorities [17].

One of the most important areas of risk of intestinal parasitic infections in the community is the risky nature of some jobs. These conditions facilitate the transmission of disease through the close contact with the infectious sources that are an integral part of some of the jobs, so they provide the possibility of easy infection [18]. The production and sales of health food products are always a concern in many developed and developing countries. Today, a number of factors including existence of quality control procedures, strict supervision of production processes, reduction in the amount of contact with food, and food categories in the process of production are the most important tasks in the production of high quality products. In this case, the role of food handlers, as the final agent of product provider, is very important because they can make unsafe and hazardous foods for consumption. If these people fail to follow the principles of primary health care and personal hygiene, they are considered as one of the main sources of transmission of pathogens through food and can easily pass many infectious agents, especially intestinal parasites to their customers [18, 19]. Obviously, the study of parasitic diseases among employees of the food industry and the prevalence rate of infection in them will help to reduce and control the spread of these diseases. Since our country has several areas with different climatic diversity and social and cultural patterns, study of parasitic infections in any province or region is absolutely necessary.

Given the role of food handlers in the spread of parasitic diseases and also due to the prevalence of infection in the country and lack of awareness towards the infection level in the city of Tabriz, this study was conducted to investigate the intestinal parasitic contamination in food handlers in the city of Tabriz in 2014.

## 2. Materials and Methods

According to existing regulations, Health Care Card is necessary to monitor the health status of employees in the health sector of different jobs and especially jobs that were related to food. All those who were responsible for procurement, distribution, and sale at food centers have referred to health centers to extend their Health Care Cards twice per year. The food handlers should undergo the necessary tests, especially parasitic tests, to receive the Health Care Card. For each food handler, 3 stools were tested by direct method (wet method with Gram) and the results were recorded along with other personal information such as age, sex, and address in the offices for each job. According to the Office Inspection Act of Tabriz, everyone is required to obtain a Health Care Card and undergo test for parasites. In this study, the recorded data related to epidemiological intestinal parasitic infection of individuals who are working at procurement, distribution, and sale of food products in public places of Tabriz in 2014 were used. In addition, other data including details of the type of business, business location, gender, and the results of tests were obtained from these offices. Based on the present data, Tabriz city had 1465 center for sales and distribution of food in 2014 and a total of 4612 person were working at these centers. The results of tests were analyzed by Spss version 18 (IBM, USA) using the chi-square test and t-test.

## 3. Result

The results showed that 3966 male food handlers (85.99%) and 646 female food handlers (14.01%), who were engaged in the distribution and sale of food, have been referred to health centers to achieve the Health Care Card. Their ages were between 14 and 68 years. The education level of the food handler was observed to be under high school diploma (30.68%), high school diploma to B.S. (58.2%), and over B.S. (11.12%).  Table 1 shows the number of positive cases according to age, sex, and education level. There was no significant difference between age and gender in any case. The infection was observed in 156 cases (3.38%) of males and 16 cases (0.345%) of females but statistically significant difference was not observed in this field (*P* = 0.094).

Parasitic comparison between the food suppliers is given in Table 2, indicating that the highest percentage of infection is related to restaurants and supermarkets, but in general, there was no statistically significant difference between different professions (*P* = 0.112). A total of 172 subjects (3.73%) were diagnosed with intestinal parasites and five types of intestinal parasites;* Giardia lamblia*,* Entamoeba coli*,* Ascaris*,* Entamoeba histolytica*, and* H. nana* were observed. Most of the infections were related to* Giardia* with 109 (63.33%) of 172 positive cases.* Entamoeba* coli infection was recorded as 38 positive cases (22.1%), while* Ascaris* infection was 10 cases (5.83%). Furthermore, the parasitic worm infection was related to* Ascaris* and* H. nana* and equal to 16 cases (0.38%) but protozoa infections were 156 cases (3.38%).

## 4. Discussion

The result of this study showed that 3.73% of the food handlers in Tabriz were infected with intestinal parasites. The parasitic infections in this study were less than the results of other studies which have been conducted in Yazd [10], Varamin and Sanandaj [20, 21], and Hamadan [22]. These studies have been conducted in the past decade and, as it was stated, show high amounts of parasitic infection, but in the present decade their prevalence in different areas has significantly declined parallel to improvement of public health. One of the most effective methods in reducing parasitic infection was strict enforcement of sanitary regulations in recent years, so that all people in Tabriz were issued a Health Care Card and parasitological tests were performed on them accurately.

The highest prevalence of infection was in supermarket and sandwich and pizza businesses which recorded 22.1, 8 and 19.2% of parasitic infections, respectively. The case study conducted in Kerman by Salary and Safizadeh is fully in accordance with the results of our study, so that the maximum amount of infection was 27.1% which was recorded for supermarket workers and clerks among the food handlers of Kerman [11]. Also, the prevalence level was reported to be 30.2% in another survey that was conducted by Rouhani et al. in Nowshahr and Chaloos on various food vendors [23]. Koohsar et al. have also reported that intestinal parasitic infection in food handlers of Gorgan was equal to 6%. According to these observations, most parasitic infections were caused by butchery staffs by 25% and the highest rate of infection was due to the parasite* Giardia* [24]. The difference between the results could be due to the sample size, the studied population, social, economic, and geographical characteristics, and climate changes, including humidity and heat, direct exposure to the sun's rays, and a lack of vegetation. In another study, the reason for these differences is highlighted [25]. Kamau et al. have conducted a similar study in Kenya and they found out that* Giardia* parasite is one of 6 common types of parasites among members of restaurant staff [26].

Although there is no direct contact with food by supermarket worker as other businesses but the contamination of food are possible when they are uncovered; therefore, sandwich, pizza, and restaurant handlers have most contribution in incidence of parasitic infection after supermarkets, which is accordance with most studies in the our country. Since these groups have direct contact with food, intestinal parasitic infection is more possible; thus the staff in these sites require personal and professional hygiene in their work environment, and these health professionals are decisively required to follow up and monitor. In this study, most parasitic infections are related to* Giardia* with 109 of the 172 positive samples which assigned 63.37% of the total parasitic infection that are well correlated to the study that took place in the city of Kerman by Salary and Safizadeh but the* Giardia* parasite has been isolated only in their study which is considered as difference with our study [11]. In another study, the prevalence of intestinal parasites was recorded in food handlers involved in the procurement, distribution, and sale of food in public places in Gilan Province and it was detected that the most common parasite is* Giardia* [9]. The studies conducted in Saudi Arabia and Sudan cleared that* Giardia* and* E. histolytica* parasites were observed in food handlers and the amount of* Giardia* parasite was very high in Sudan [27, 28].

In this study, the rate of infection with worms was 16 samples (0.345%) and protozoan infection was at the rate of 156 samples (3.38%). These findings are in line with the earlier studies conducted by Kheyrandish et al. in Khorramabad, Shidfar et al. in Ilam, and Balarak et al. in Ahar [29–31]. Also, a correlation was found with a study in Ethiopia and India [32, 33]. These studies show higher prevalence of protozoan parasites than worms. The high prevalence of protozoan parasites is due to the proliferation of the spread and ability to produce large simple cysts and protozoa stability in environmental conditions [34]. In the study conducted in Nigeria and Manila, protozoan parasites like* Giardia* and* Entamoeba* coli were more observed, due to the higher proliferation of protozoa than worms [35, 36]. According to various studies, it is indicated that the prevalence of intestinal parasitic infections has recently changed compared to the past and the prevalence of infection has declined, totally. The reduction in the incidence of infections over the years can be attributed to the development of networks for the distribution of drinking water, more comprehensive monitoring of health systems, and ongoing communication with employees and stricter rules than in the past to provide health advice and provide hygiene standards, continuous testing of parasitic infections (6 months) and the availability of drugs for the treatment of infections, higher levels of life expectancy in terms of health and increasing level of individual health information and the use of less human fertilizer by farmers [19, 37].

Literacy level reduces the number of positive samples; in other words, there is a significant relationship between level of education and degree of parasitic infection (*P* = 0.0044). It could be interpreted that if the literacy rate increased, then awareness about parasitic infections will also increase. Therefore, the lower need for health advice and better compliance with sanitary regulations will be achieved, as noted in other studies [29]. People in South-East Asia have less knowledge about these infections; thus, there are more infections in those regions [38], while the infection level is less in developed countries like Italy [39].

## 5. Conclusion

The results show that the prevalence rate of intestinal parasites, particularly* Giardia* protozoan, in food handlers is 3.73%. This percentage of infection between the workers in food producing places can be considered as an important way to transmit and incidence of these infectious agents among the public. Therefore, the attention to the health status of these places and the continuous monitoring of the workers in these places can play significant role to prevent the infection. Furthermore, holding of training and educational programs leads to increase the knowledge of the workers towards these infections and the associated risk to themselves and their community which can be assumed as other effective factors to diminish the health risk problems associated with this sector.

## Figures and Tables

**Table 1:** The number of positive cases of parasites in food production and distribution centers employees in the city of Tabriz according to individual characteristics.

Characteristics	Studied people		Positive cases	
	Numbers	%	Numbers	%
Gender				
Male	3966	85.99	156	3.38
Female	646	14.01	16	0.345
Age (year)				
Under 20	418	9.05	20	0.433
20 to 40	2576	55.85	108	2.34
Over 40	1618	35.1	44	0.954
Education				
Under high school diploma	1415	30.68	68	1.75
High school diploma to B.S.	2684	58.2	76	1.64
Over B.S.	513	11.12	28	0.607

**Table 2:** Frequency of pathogenic intestinal parasites on the separation of producers and suppliers of food trade in Tabriz.

Pollution trade unit	Total	Without infection	Infection							
			*Giardia*	*Ascaris*	*Entamoeba coli*	*H. nana*	*Entamoeba histolytica*	Number of positive samples	% of positive samples	% of all samples
Supermarket	880	842	23	4	7	2	2	38	22.1 (38/172)	0.823 (38/4612)
Restaurant	821	797	14	2	5	1	2	24	13.95 (24/172)	0.52 (24/4612)
Sandwich and pizza	709	676	21	2	8	1	1	33	19.2 (33/172)	0.715 (33/4612)
Confectionery	229	221	5	—	2	—	1	8	4.65 (8/172)	0.173 (8/4612)
Bakery	274	265	5	1	2	1	—	9	5.25 (9/172)	0.195 (9/4612)
Butcher	95	91	2		1	—	1	4	2.32 (4/172)	0.086 (4/4612)
Dairy	403	383	13	1	5	—	1	20	11.65 (20/172)	0.433 (20/4612)
Production workshop	889	876	11	—	2	—	—	13	7.55 (13/172)	0.281 (13/4612)
Ice cream and juice	312	289	15	—	6	1	1	23	13.3 (23/172)	0.498 (23/4612)
Sum	4612	4440	109	10	38	6	9	172	—	—
% of positive samples	—	—	63.37	5.81	22.1	3.48	5.23	100	100	—
% of all samples	100	96.27	2.36	0.0021	0.0082	00.13	00.195	3.73	—	3.73
